# An Investigation into the Adsorption Mechanism of Organic Anions on a New Spandex

**DOI:** 10.3390/polym14153108

**Published:** 2022-07-30

**Authors:** Xiaoxing Shen, Pu Gao, Tingting Jin, Yi Ding, Chaoyan Bao

**Affiliations:** College of Advanced Materials Engineering, Jiaxing Nanhu University, Jiaxing 314001, China; shenxiaoxing2@126.com (X.S.); jintingting0325@163.com (T.J.); xiaoding0518@126.com (Y.D.); baochaoyan0926@163.com (C.B.)

**Keywords:** dyeable spandex, structure, neutral red G, adsorption kinetics, adsorption thermodynamics

## Abstract

In recent years, there has been significant interest in the study of spandex in high-elasticity sensors. As a new kind of special spandex, dyeable spandex shows strong adsorption capacity for anions. In this study, neutral red G was used as an anion adsorption simulator to study the adsorption mechanism of dyeable spandex on anionic materials. The structure of dyeable spandex was characterized by the modern instrumental analysis method, and the adsorption kinetics and thermodynamics of neutral red G on dyeable spandex were discussed. The results show that the use of mixed amines as chain extenders for dyeable spandex reduced the regularity of molecules and the crystallinity of spandex, which was beneficial to the diffusion adsorption of anions. On the other hand, the number of secondary amino groups increased, providing more adsorption sites under acidic conditions. The adsorption of neutral red G on dyeable spandex satisfied the quasi-second-order kinetics and the Langmuir adsorption model, indicating that dye adsorption on spandex was mainly electrostatic. The diffusion coefficient and equilibrium adsorption capacity of neutral red G on dyeable spandex increased significantly, whereas enthalpy and entropy decreased.

## 1. Introduction

Due to its excellent elasticity, spandex integrates the comfort, function, and beauty of textiles and has become an important raw material in the textile industry’s development. As a result, spandex applications have rapidly expanded from traditional textiles to emerging fields such as sports, medical, sanitary, and quartermaster products [1,2,3,4]. Moreover, in recent years, due to the influence of intelligent wearable electronic products, the research boom of intelligent electronic textiles has risen in the textile field, resulting in high demand for conductive sensing materials with high flexibility and elasticity [5,6,7,8]. As a result, the development of textile-based semiconductor materials has become a focal point of contemporary research. Thus, due to its high elasticity, spandex has attracted the attention of researchers. As a result, it is considered an ideal load matrix and shows a broad application prospect.

Ordinary spandex often lacks functional groups that can be combined with conductive materials [9]. Song [10] found that multi-walled carbon nanotubes and oxidized graphene were coated on the surface of ordinary spandex by electrostatic layer-by-layer self-assembly, and then oxidized graphene was reduced by hydrazine hydrate to obtain conductive spandex, which was used in wearable flexible sensors, but the conductivity decreased with the increase in washing times. Therefore, the key to resolving this issue is the development of spandex containing a large number of functional groups that can be combined with conductive materials. Therefore, the key to resolving this issue is the development of spandex containing a large number of functional groups that can be combined with conductive materials.

In recent years, acid-dyeable spandex, such as H550 by Korea’s Hyosung, has been developed. This kind of spandex requires special spinning materials and processes. Moreover, the prepared spandex contains many functional groups that can provide reaction points for combining dyestuffs and conductive materials. However, studies on the adsorption mechanism of graphene oxide, carbon nanotubes and other conductive materials containing certain anions on dyeable spandex are still limited [11,12,13,14,15,16,17,18]. The present study used anionic dye instead of anionic conductive material to investigate the adsorption performance of dyeable spandex. On the one hand, dyes are cheaper, more readily available and more stable than graphene. On the other hand, dyes have bright and stable visual characteristics, which is convenient to observe the adsorption results of anions on dyeable spandex. Therefore, in this study, neutral red G was used as an anion adsorption simulator to study the adsorption mechanism of dyeable spandex on anionic materials. The adsorption kinetics and thermodynamics of neutral red G on dyeable spandex were also discussed. The research results have important implications for the development of flexible wearable sensors with dyeable spandex as a conductive substrate.

## 2. Experimental

### 2.1. Materials

Hyosung (Seoul, Korea) supplied the spandex types (40 deniers), 4700 (dyeable spandex produced by Hyosung Spandex (Korea Gumi) Co. LTD), and 4889 (ordinary spandex produced by Hyosung Spandex (Jiaxing) Co. LTD). Before dyeing, the spandex fabric was scoured using 0.2 g/L NaOH and 1 g/L scouring agent (MCH-125CC) at 80 °C for 20 min.

### 2.2. Color Depth Measurement for Dyed Spandex

The K/S value is a constant, K represents the color absorption coefficient, S represents the color scattering coefficient, and this ratio has a linear relationship with the dyeing depth C (non-linear). Usually, the K/S value is used to represent the color depth of the dyed object. The higher the K/S value, the darker the color. The color depth of dyed spandex fiber was evaluated by measuring the K/S value. A Datacolor SF600 colorimeter was used under a D65 light source and a 10° viewing angle. The measurement was repeated four times, and the average value was obtained.

### 2.3. Thermogravimetric Analysis

The dyeable spandex and ordinary spandex fibers were cut into powder using a Hastelloy slicer. Then, the Mettler Toledo TGA/DSC 3+ synchronous thermal analyzer (Columbus, OH, USA) was used to measure the TG curve of the samples under a nitrogen atmosphere with a heating rate of 10 °C/min over a temperature range of 20–800 °C. The heat resistance of the two fibers was analyzed, and the crystallinity of spandex was deduced.

### 2.4. Fourier Transform-Infrared Spectroscopy

After being placed in the oven at 105 °C for 4 h, both ordinary spandex and dyeable spandex were ground in a Wiley mill. In addition, Fourier-Transform Infrared (FTIR) spectra of both fibers were then taken on a Nicolet iS50 spectrophotometer (Thermo Fisher Scientific, Waltham, MA, USA) using Kalium Bromatum (KBr) pellets containing 1% of finely ground samples. Each spectrum was scanned 32 times across the 4000–400 cm^−1^ range. The dyeable spandex characteristic groups were investigated, and the dyeing mechanism of dyeable spandex was hypothesized based on the FTIR spectra.

### 2.5. H NMR Test

Fifteen milligrams of dyeable spandex and ordinary spandex were dissolved in deuterium DMF. The samples were then tested using a Brucker 600 MHz nuclear magnetic spectrum (Billerica, MA, USA) with a scanning power of 600 M and a scanning time of 128 times. The spandex structure was analyzed using NMR spectra, and its dyeing mechanism was studied.

### 2.6. Determination of Adsorption Capacity

#### 2.6.1. Experiments of Adsorption

**Figure 1:**
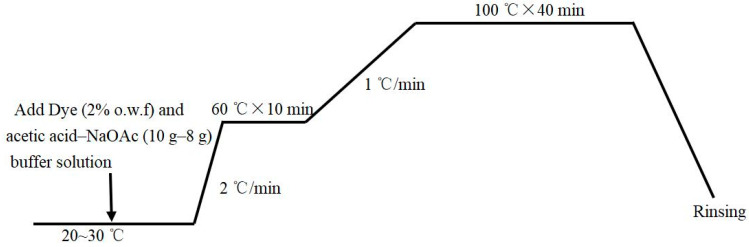
The dyeing process.

Neutral red G was provided by Shanghai Anoky group Co., Ltd. (Shanghai, China), and Isolan yellow NHF-S and Isolan black 2S-EL were provided by Dystar (Shanghai, China) Trading Co. They were applied to dye the fabrics separately in the traditional way for nylon in an IR DYER infrared testing machine produced by Xiamen Rapid Precion Machinery Co., Ltd (Xiamen, China). Acetic acid and sodium acetate produced by Sinopril Chemical Reagents Co., Ltd (Shanghai, China). were used in the dyeing process to adjust the pH of the dyeing bath to 4.5. The whole dyeing process is shown in Figure 1.

#### 2.6.2. Experiments of Adsorption Kinetics and Thermodynamics

##### Calculation of Equilibrium Adsorption Capacity, Half Dyeing Time and Rate Constant

Drawing Standard working curve: 10 g/L of neutral red G was prepared, and then 1 mL, 2 mL, 4 mL, 6 mL, 8 mL, and 10 mL, consecutively, were poured into a 100 mL volumetric flask. Then, a spectrophotometer U-3900/3900H was used to measure the maximum adsorption wavelength λ_Max_ of neutral red G. Afterward, the absorbance of the different dyestuff concentrations at λ_Max_ was measured. The standard working curve of neutral red G was drawn with the mass concentration of dye as abscissa and the absorbance as ordinate. Next, the concentration of the dye solution was calculated using the standard working curve. Finally, the dyeing amount of neutral red G on the fiber was determined.

Measuring the dyeing rate curve: the spandex was dyed at 100 °C for 5 min, 10 min, 15 min, 20 min, 30 min, 40 min, 50 min, 60 min, 80 min, 100 min, 120 min, 140 min, respectively, in a dye bath of liquor ratio of 100:1 using 1 g/L acid leveling agent and maintaining a pH of 4.5 (acetic acid-sodium acetate: 10–8 g) [19,20,21,22,23]. The dye concentration was 2% (o.w.f). The absorbance of the dyeing liquid was measured to determine the dye concentration on the fiber. The dyeing rate curve was drawn according to the dye concentration and holding time relationship. According to the curve, the dyeing kinetic parameters such as the equilibrium adsorption capacity, time of half dyeing, and dyeing rate constants were calculated using Formulas (1) to (4).

(1) $$\frac{t}{C_{t}}=\frac{1}{C_{\infty }}t+\frac{1}{kC_{\infty }^{2}}$$

(2) $$C_{\infty }=\frac{1}{tg\partial },K=\frac{1}{bC_{\infty }^{2}}$$

(3) $$t_{1/2}=\frac{1}{KC_{\infty }}$$

(4) $$\begin{array}{c} \frac{M_{t}}{M_{\infty }}=1-4\overset{\infty }{\underset{n-1}{\sum }}\frac{e^{-V_{n}D_{t}/a^{2}}}{V_{n}^{2}}=\\ 1-4\{\frac{1}{5.785}e^{-5.785D_{t}/a^{2}}+\frac{1}{30.47}e^{-3047D_{t}/a^{2}}+\frac{1}{74.89}e^{-74.89D_{t}/a^{2}}+\frac{1}{139}e^{-139D_{t}/a^{2}}+\cdots \cdots \} \end{array}$$

where C_∞_ is the dyestuff concentration on the spandex fiber at dyeing equilibrium, C_t_ is the concentration at time t, K is the dyeing rate constant, and t_1/2_ is the time of half dyeing.

##### The Quasi-First-Order Kinetic Model

The quasi-first-order kinetic model adopts Lagergren’s first-order rate equation based on solid adsorption volume, which is the most common and is used to study the adsorption kinetic equation of solute adsorption on a solid adsorbed substance in the liquid phase. The calculation refers to Formula (5). If the reaction follows first-order kinetics, a straight line is obtained by plotting t against ln (q_e_ − q_t_):

(5) $$In\;(q_{e}\;-\;q_{t})=In\;q_{e}-k_{1}t$$

##### The Second-Order Kinetic Model

The second-order kinetic model assumes that the adsorption rate is controlled by the chemical adsorption mechanism, which involves electron sharing or electron transfer between the spandex and the dyestuff. The calculation is based on Formula (6).

Where t/q_t_ is used to plot t. If a straight line is obtained from linear fitting, it indicates that the adsorption reaction conforms to the second-order adsorption kinetics model. The degree of compliance can be determined by the judgment coefficient R^2^ of data linear fitting.

(6) $$\frac{t}{q_{t}}=\frac{1}{k_{2}q_{e}^{2}}+\frac{1}{q_{e}}t$$

##### Thermodynamics of Adsorption

Adsorption isotherms of dyes on spandex: the spandex was successively dyed at 80, 90, and 100 °C for 180 min in a dye bath of 100:1 liquor ratio using a 1 g/L acid leveling agent and maintaining a pH of 4.5 (acetic acid-sodium acetate). The dye concentration of spandex at each dyeing temperature was 1, 2, 4, 6, 8, and 10% (o.w.f). When the spandex had finishing being dyed, the absorbances of the dyeing residual solution were measured, the dye concentration in the residual solution and the dye concentration on the spandex fiber were calculated, the adsorption isotherm was drawn according to the dyestuff concentrations of solution and fiber, and △G°, △H°, and △S° were calculated using Formulas (7) to (12).

(7) $$\frac{C_{e}}{q_{e}}=\frac{1}{Qb}+\left(\frac{1}{Q}\right)C_{e}$$

(8) $$\frac{1}{q_{e}}=\frac{1}{Q}+\frac{1}{QbC_{e}}$$

(9) ΔG°=−RTIn(b)

(10) $$In\left(\frac{b_{2}}{b_{1}}\right)=-\frac{\Delta H{}^{\circ}}{R}\left(\frac{1}{T_{2}}-\frac{1}{T_{1}}\right)$$

(11) ΔG°=ΔH°−TΔS°

(12) $$Inq_{e}=InQ_{f}+\frac{1}{n}InC_{e}$$

where q_e_ is the amount of dye adsorption on the fiber at dyeing equilibrium; C_e_ is the dye concentration in dyeing solution at dyeing equilibrium; Q is the amount of dye formed by complete monolayer adsorption on the fiber surface at equilibrium; b is the constant associated with localization adsorption.

If dyeing thermodynamics conforms to the Langmuir adsorption isotherm, Formulas (7) to (11) are used to calculate thermodynamic parameters. Additionally, if it conforms to the Freundlich model, Formulas (9) to (12) are used to calculate thermodynamic parameters.

## 3. Results and Discussion

### 3.1. Dyeing Properties of Dyeable Spandex

**Figure 2:**
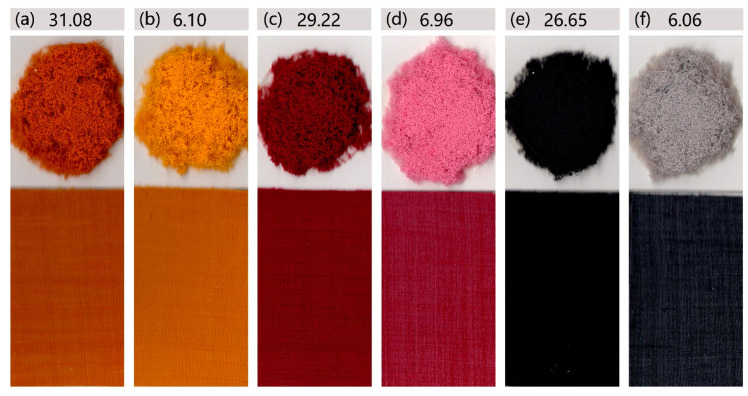
Comparison of dyeing properties between dyeable spandex and ordinary spandex: (**a**,**c**,**e**): dyeable spandex and fabric woven from dyeable spandex and Nylon 6 dyed with yellow, red, and black dyestuffs, respectively; (**b**,**d**,**f**): ordinary spandex and fabric woven from ordinary spandex and Nylon 6 dyed with yellow, red, and black dyestuffs, respectively.

The dyeing results and K/S value of neutral red dyestuffs on dyeable spandex and ordinary spandex are shown in Figure 2.

The dyeing depth of dyeable spandex was far deeper than that of ordinary spandex, which showed better dyeing performance. For example, after dyeing with neutral red G, the K/S value of dyeable spandex was 31.08, whereas the color of ordinary spandex was lighter, only 6.10. Similarly, when the spandex was dyed with yellow dyestuff, dyeable spandex was 29.22 and ordinary spandex was 6.96 When dyed with black dyestuff, dyeable spandex was 26.65 and ordinary spandex was 6.06. Meanwhile, after dyeing circle knitted fabric made of spandex and nylon, the fabric color of dyeable spandex was darker than the fabric made of ordinary spandex and nylon, especially black, using the same dyeing process. Therefore, spandex-rich fabrics can be woven with dyeable spandex, which can solve the problem of grin through.

### 3.2. Analysis of Spandex Chemical Structure

The chemical structure of the fiber determines its physical and chemical properties, such as its dyeing and mechanical properties. In order to study the dyeing mechanism of dyeable spandex, the chemical structures of dyeable spandex and ordinary spandex were analyzed by Thermogravimetric analysis, FTIR spectrometer, and ^1^H NMR test.

#### 3.2.1. Thermogravimetric Analysis

**Figure 3:**
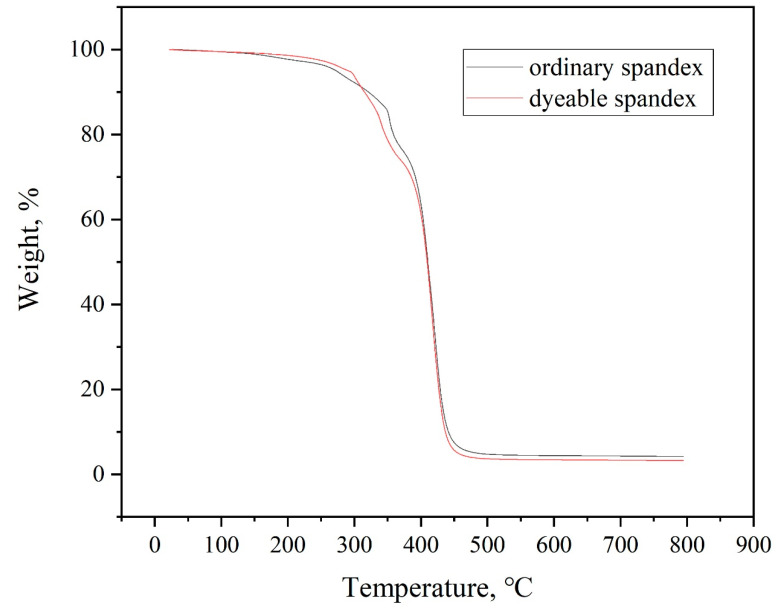
TG curves of dyeable spandex and ordinary spandex.

**Figure 4:**
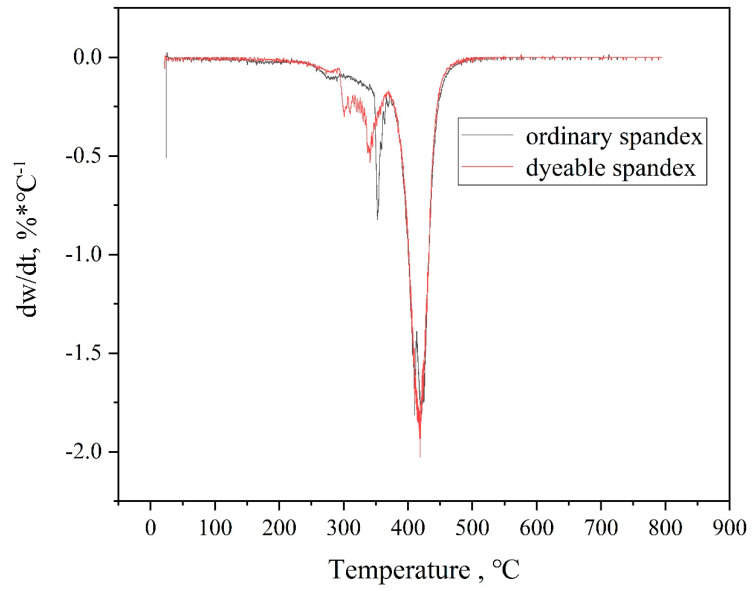
DTG curves of dyeable spandex and ordinary spandex.

The thermodynamic properties of fiber directly impact their dyeing and finishing processes, as well as their performance. Additionally, as the temperature rises, the interaction between molecular chains weakens. Thus, when the temperature reached a certain point, the crystallization area within the fiber reduced, and the amorphous area increased, which was conducive to the dyeing of spandex. Figure 3 and Figure 4 depict the TG and DTG of ordinary spandex and dyeable spandex, respectively.

Figure 3 shows that the thermal stability of dyeable spandex was slightly lower than ordinary. The initial cracking temperature of dyeable spandex was about 280 °C. Correspondingly, that of ordinary spandex was about 350 °C, and with the increase in temperature, the weight loss of the dyeable spandex was always greater than ordinary spandex. This indicates that as the temperature increases, more chain segment thermal cracking occurs in the dyeable spandex. As shown in Figure 4, the weight-loss rate of dyeable spandex is faster than that of ordinary spandex. The first weight-loss zone of the dyeable spandex appeared at 280 °C. When the temperature reached 420 °C, the weight loss rate of dyeable spandex reached its maximum value. The thermal stability of the dyeable spandex was lower than that of ordinary spandex. This was mainly due to the use of special soft chain raw materials such as n-methyl-2,2-diaminodiethylamine, ethylenediamine, propylene diamine, hexediamine, 2-methylpentanediamine and others in dyeable spandex. The introduction of these mixed-amine chain segments has reduced the linear regularity of soft connecting segments, which reduced the crystallinity of the synthetic fiber, increased the proportion of amorphous area, and caused the molecular structure to become fluffy. Thus, changes in the structure of dyeable spandex lead to a decrease in the thermal stability of dyeable spandex while increasing the dyeability of the fiber.

#### 3.2.2. Fourier Transform-Infrared Spectroscopy

The structure of dyeable spandex and ordinary spandex was analyzed using an FTIR spectrometer. Figure 4 depicts the FTIR spectra of dyeable spandex and ordinary spandex.

**Figure 5:**
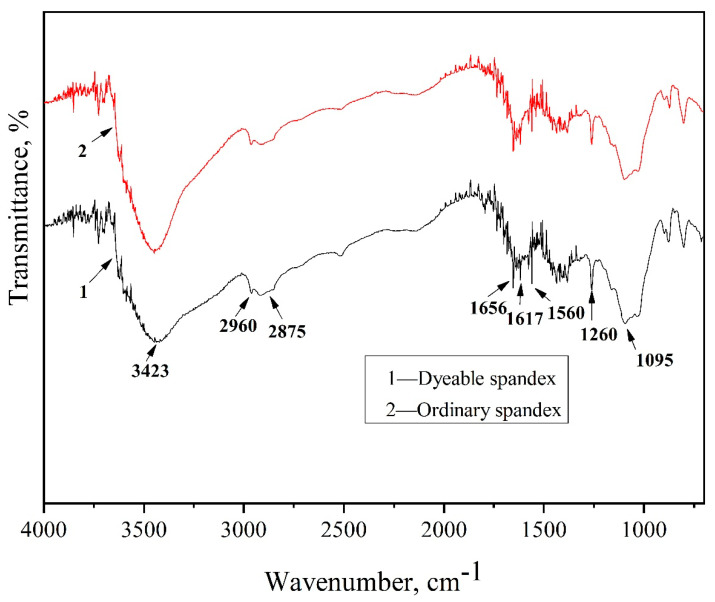
Infrared spectrum of dyeable spandex and ordinary spandex.

Figure 5 shows that the stretching vibration peak of N-H that forms hydrogen bonds was 3423 cm^−1^ in the spectrum. The stretching vibration peaks of methyl and methylene C-H were 2875~2960 cm^−1^. Additionally, the stretching vibration peaks of carbamate -C=O were 1656 cm^−1^ and 1617 cm^−1^, respectively. The aminoester amideⅡband formed by the combination of -N-H deformation vibration and -C-N stretching vibration was 1560 cm^−1^, and 1260 cm^−1^ was the aminoesteryl amide Ⅲ band formed by the coupling of -N-H deformation vibration and -C-N stretching vibration. In addition, a comparison of infrared spectra of the two fibers showed that the adsorption peak area of dyeable spandex at 3423 cm^−1^ was larger than that of ordinary spandex, indicating that the dyeable spandex contained a large amount of secondary amine -N-H. Meanwhile, the adsorption peak area at 1560 cm^−1^ of dyeable spandex, which is the deformation vibration peak of -N-H, was obviously stronger, indicating that -N-H increased. Under acidic conditions, -N-H could adsorb H^+^ and bind with the sulfonic acid and carboxylic acid of the acidic dye, greatly improving the spandex dyeing rate. Therefore, it can be seen from the infrared spectrum that there was a large amount of secondary amine -N-H in dyeable spandex, which provides a “dye block” for dyestuffs, greatly improving the dyeing property and resulting in a better dyeing adsorption effect.

#### 3.2.3. H NMR Analysis

To better research the dyeing mechanism of dyeable spandex, the chemical structure of spandex was analyzed using ^1^H NMR.

**Figure 6:**
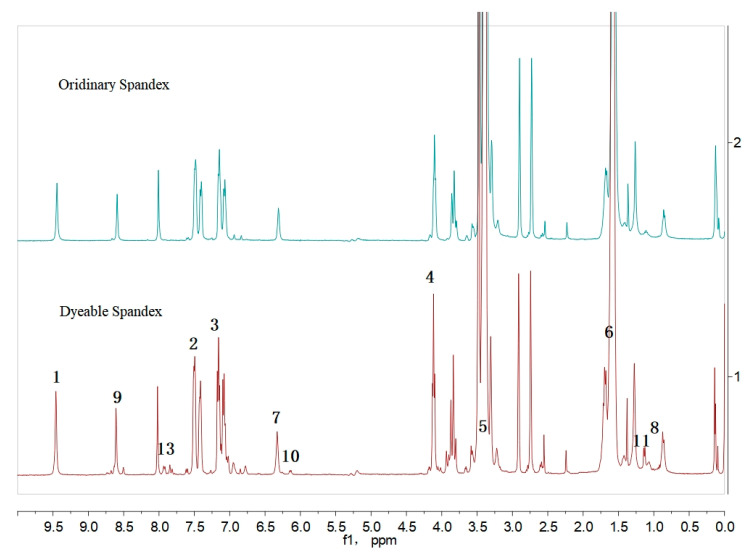
^1^H NMR spectrum of spandex fiber.

According to prior studies [24,25], peaks near 8.03 ppm and 2.92 ppm, as shown in Figure 6, were solvent adsorption peaks of deuterium DMF. Additionally, according to chemical shift, the peaks of 1, 2, 3, and 4 were for spandex hard chain segment 4,4’-diphenylmethane diisocyanate (MDI) hydrogen atoms; 1 was for hydrogen of amino on the links of the benzene ring; 2 and 3 were for the hydrogen adsorption peak of a benzene ring; 4 was for the methyl hydrogen adsorption peak between two benzenes. In addition, 5 and 6 were the adsorption peaks of hydrogen on the alkyl group of polytetrahydrofuran (PTMG) in the soft connecting segment of spandex; 3.52 ppm was the hydrogen connected with oxygen atoms; 1.77 ppm was the hydrogen peak on the alkyl group without electron adsorption on both sides. In addition, 7, 8, and 9 were adsorption peaks of corresponding hydrogen atoms on chain extender ethyleneamine (EDA). The above adsorption peaks existed in the NMR spectra of both dyeable spandex and ordinary spandex. The adsorption peaks of dyeable spandex are as follows: 9, 10, 11, and 12 peaks, which are the adsorption peaks of hydrogen on chain extender 1, 2-propylene diamine (PDA). Thus, two chain extenders were used in dyeable spandex. Propylene diamine linearity was lower than ethylenediamine, which increased the irregularity of polymer formation, reduced the crystallinity of spandex, and increased the amorphous area of spandex, making it more dyeable. A large number of free amino groups was also added to the chemical structure of dyeable spandex, as evidenced by the presence of adsorption peak 13 for adsorption Secondary amino groups attached to an electron-donating group such as an alkyl group. Under acidic conditions, the spandex was dyed using an ionic bond with the sulfonate of acid dye.

### 3.3. Study of Dyeing Thermodynamics and Kinetics of Dyeable Spandex

#### 3.3.1. The Standard Working Curve of Neutral Red G

**Figure 7:**
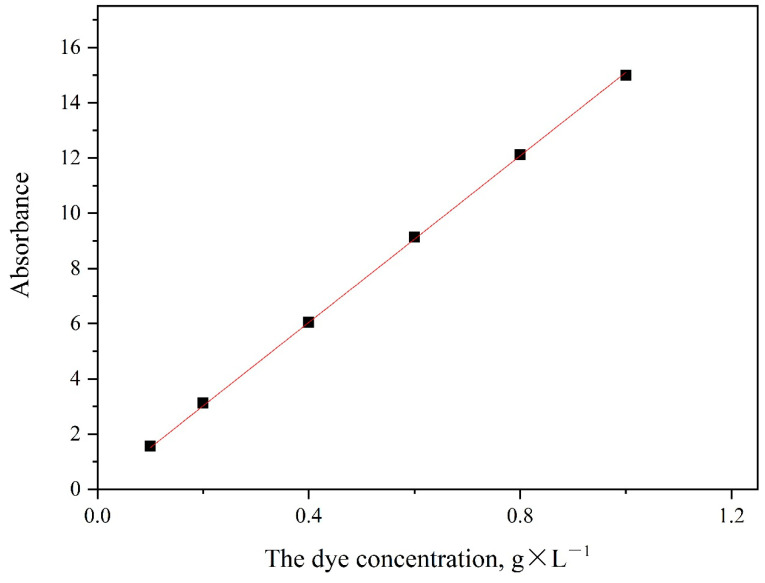
The standard working curve of neutral red G.

According to Lambert Beer’s law, within a certain dyestuff concentration range, the concentration C of a dye solution is proportional to the absorbance A measured by a spectrophotometer. For example, the λ_max_ of neutral red G was 492 nm, and as shown in Figure 7, the standard working curve can be obtained by measuring the matched standard solution at the maximum adsorption wavelength.

By fitting the experimental data with Origin 2018(64 Bit) software developed by OriginLab, the linear equation of neutral red G dye was as follows:

(13) y=15.00427x+0.06954  R2=0.99987

where x is the mass concentration of neutral red G, and y is the absorbance A.

Drawing the standard working curve was the prerequisite for studying dyeing kinetics and thermodynamics. Fitting the experimental data yielded an R^2^ of 0.99987, indicating that the dye mass concentration was positively correlated with the absorbance A value and that the accuracy of the working curve met the requirements.

#### 3.3.2. Dyeing Kinetics of Spandex Fibers Dyed with Neutral Red G

##### Dyeing Rate Curve

**Figure 8:**
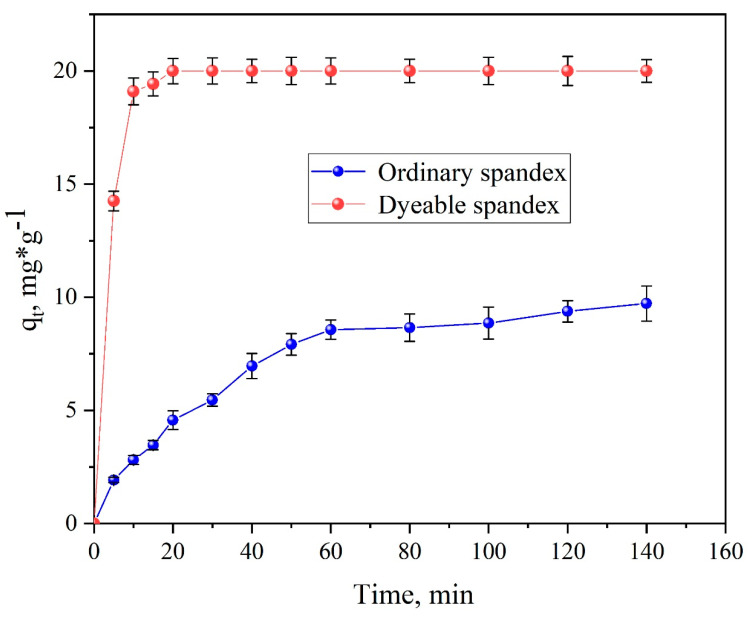
Dyeing rate curves of neutral red G on two kinds of spandex.

Figure 8 shows that when dyeable spandex was dyed with neutral red G, the initial dyeing rate was very high and reached 97.25% after 15 min. After the holding time reached 20 min, the dyeing process reached equilibrium. However, the initial dyeing rate and equilibrium dyeing amount of ordinary spandex were obviously lower than those of dyeable spandex, and the equilibrium state was still not reached when the dyeing time was extended to 120 min. The reason for the obvious difference is that the neutral red G was mainly bonded with the fiber through the intermolecular van der Waals forces and hydrogen bond when dyeing the ordinary spandex, whereas the dyeable spandex was mainly bonded with the fiber through electrostatic attraction in addition to the intermolecular van der Waals force and hydrogen bond.

###### Calculation of Equilibrium Adsorption Capacity, Time of Half Dyeing, and Rate Constant and Kinetic Model

**Figure 9:**
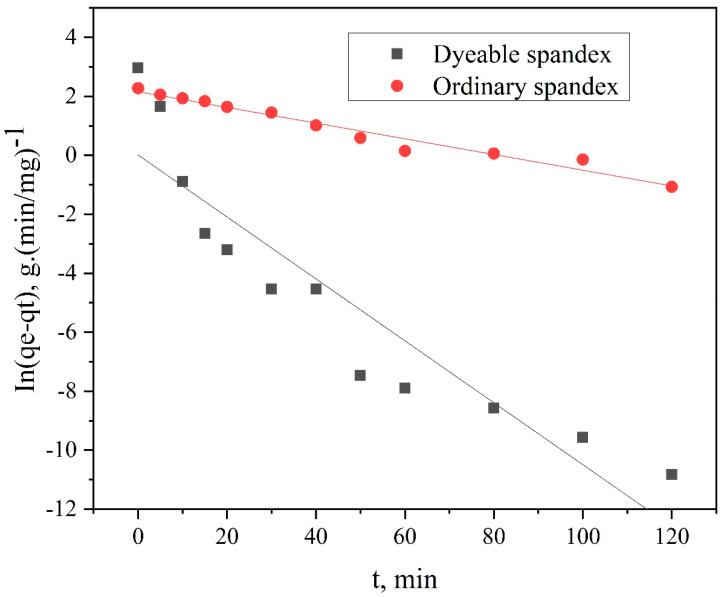
Pseudo–first-order kinetic model of neutral red G dyeing spandex.

**Figure 10:**
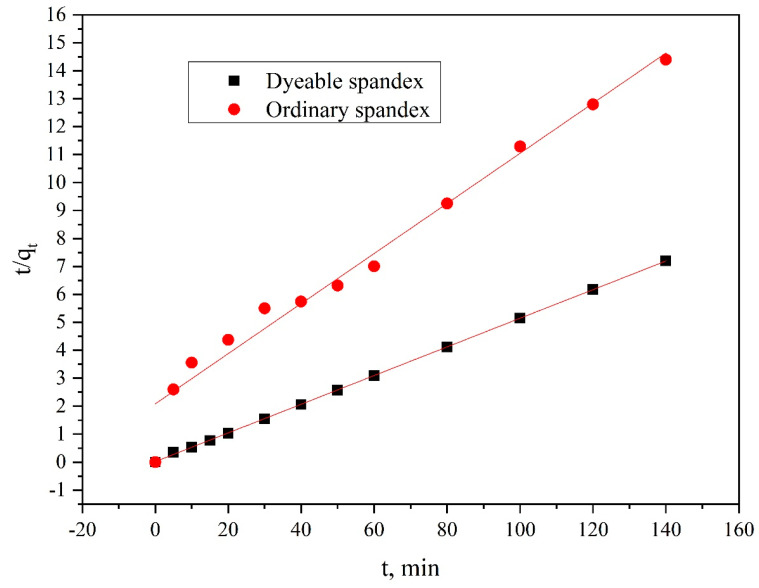
Pseudo–second-order kinetic model of neutral red G dyeing spandex.

Using Formulas (5) and (6), the kinetic model of spandex dyed was analyzed, the results as shown in Figure 9 and Figure 10, and the adsorption mechanism was speculated.

The t/C_t_ − f (t) relation curve was calculated using Formula (1), as shown in Figure 10. From the two kinetic models in Figure 9 and Figure 10, the parameters of the kinetic model for dyeing spandex with neutral red G can be calculated using Formulas (1) to (4), as shown in Table 1.

According to Formula (5), the quasi-first-order linear fitting curve of neutral red G’s adsorption on dyeable spandex and ordinary spandex was obtained, as shown in Figure 9. It can be seen that the adsorption data of dyeable spandex deviated from the In(q_e_—q_t_) —t fitting curve during the whole adsorption stage, indicating that the adsorption of neutral red G on dyeable spandex did not conform to the quasi-first-order kinetic model. In addition, the linear fitting results can be measured by regression coefficient R^2^. Generally, the higher the R^2^ value is, the more the adsorption process conforms to the quasi-first-order kinetic equation. As can be seen from Table 1, when the first-order kinetic model was used for linear fitting of data, the regression coefficient R^2^ obtained by dyeable spandex was 0.8481, indicating a low fitting degree. All these results indicated that the quasi-first-order kinetic model could not accurately describe the adsorption kinetic characteristics of neutral red G on dyeable spandex.

According to Formula (6), k_t_ and q_e_ can be directly calculated from the intercept and slope of the t/q_e_ − t curve. The larger the slope, the smaller the q_e_; thus, the smaller the dyeing equilibrium adsorption amount. According to Formula (6), the quasi-second-order kinetic model fitting curve of neutral red G adsorption on dyeable spandex and ordinary spandex was obtained (Figure 10). It can be seen from the figure that the fitting curve of the two kinds of spandex dyeing was linear. Meanwhile, the regression coefficient R^2^ of dyeable spandex was much higher than that of the first-order kinetics model (>0.99) in Table 1; therefore, the quasi-second-order kinetics can well describe the whole dyeing process of dyeable spandex with neutral red G. Since the quasi-second-order kinetics is based on the assumption that the adsorption rate is determined by the square value of the number of adsorption vacancies on the fiber surface, and the location adsorption of neutral red G on dyeable spandex is usually caused by electrostatic adsorption effect, it can be seen that the adsorption process of neutral red G on dyeable spandex is mainly ion location adsorption. At the same time, the quasi-second-order dynamic model includes all the processes of dye adsorption to the fiber, such as external liquid diffusion boundary layer diffusion, dye adsorption on the fiber surface and dye diffusion into the fiber. Therefore, the quasi-second-order dynamic model can truly and comprehensively reflect the dynamic mechanism of dyeable spandex dyed on neutral red G.

However, ordinary spandex was dyed with neutral red G, and there was no significant difference in the fitting degree between the dyeing adsorption data and the first-order kinetic model (R^2^ = 0.9645) or second-order kinetic model (R^2^ = 0.9689), indicating that the adsorption mechanism of neutral red G on ordinary spandex and dyeable spandex was quite different. The dyeing modification of spandex greatly changed the adsorption mechanism of anion on spandex.

As can be seen from Table 2, the balance adsorption capacity of dyeable spandex is 19.5007, far greater than that of the ordinary spandex (10.9745). This was because the dyeable spandex has adopted special spinning raw materials and introduced a large number of secondary amino groups into the fiber, which was not found in the ordinary spandex. Under acidic conditions, the dye base combined with neutral dye anion was greatly increased; thus, the equilibrium adsorption capacity was higher, and the good adsorption of anion on dyeable spandex was achieved. At the same time, the dye equilibrium adsorption capacity of dyeable spandex was close to that of wool, nylon, and cotton fibers, indicating that the adsorption capacity of dyeable spandex to anions has reached the level of wool and nylon fibers, and has good adsorption capacity to anions.

##### Calculation of Diffusion Coefficient

In this paper, dyeable spandex and ordinary spandex specifications were 40D and had the same diameter. An optical microscope was used to measure the fiber radius 20 times, and the average value represented the final data. The spandex fiber radius was 74.4216 µm. When Ct/C_∞_ = 0.5 and t = t_1/2_, the diffusion coefficients are illustrated in Table 2 according to Formula (4).

Table 3 reveals that at 100 °C, the diffusion coefficient of dyeable spandex dyed with neutral red G was much greater than ordinary spandex. It indicates that the amorphous area of dyeable spandex was larger, and its molecular structure was much laxer, making it easier for the dyestuff molecules to diffuse into the fiber.

#### 3.3.3. Dyeing Thermodynamics of Spandex Fiber Dyed with Neutral Red G

The study of fiber dyeing thermodynamics includes dyeing affinity, dyeing heat, dyeing entropy, and the change of chemical potential or free enthalpy when dyeing reaches equilibrium.

##### Adsorption Isotherms

The adsorption isotherm is the distribution curve between the remaining dyestuff concentration in the dyestuff solution and the dyestuff concentration adsorbed on the fiber when the spandex dyeing reaches equilibrium at a constant temperature. From the adsorption isotherm, one can determine the distribution pattern and adsorption amount of dyestuff in the fiber phase. Thus, the adsorption performance of dyestuff on fibers is closely related to the fiber structure, dyestuff structure, and dyeing environment. Usually, acid dyes and spandex are combined by van der Waals forces, the hydrogen bond, and the Coulomb force.

**Figure 11:**
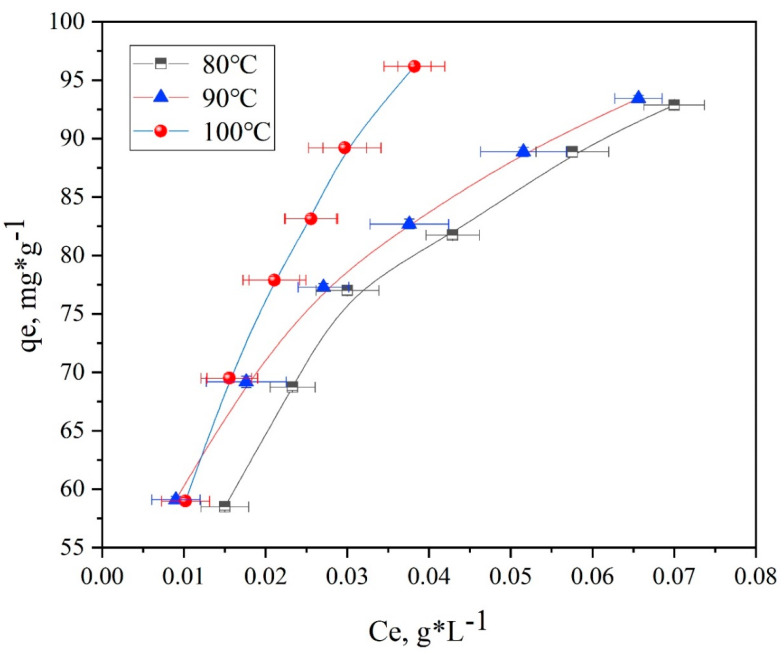
Adsorption isotherms of dyeable spandex dyed with neutral red G.

**Figure 12:**
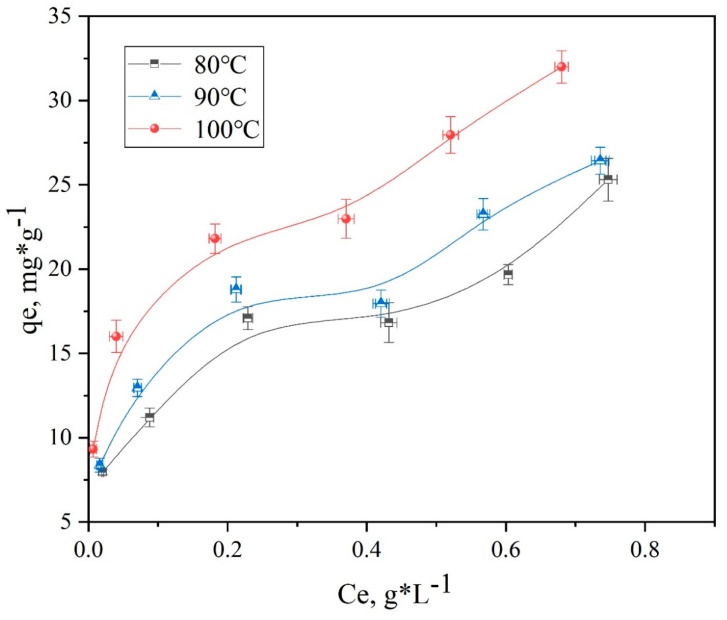
Adsorption isotherms of ordinary spandex dyed with neutral red G.

Additionally, the adsorption isotherms of spandex dyed with neutral red G at 80 °C, 90 °C, and 100 °C are shown in Figure 11 and Figure 12. However, it should be noted that the absorbance cannot be measured due to the high dyeing rate of dyeable spandex and relatively clear residual liquid. Therefore, the data points that cannot be measured for dyeable spandex were deleted.

The rate of dye uptake was significantly influenced by temperature. As shown in Figure 11 and Figure 12, the rate of dye uptake increased rapidly as the temperature rose from 80 to 100 °C. This indicated that the dyeing of spandex with neutral dyes was an endothermic reaction. At the same temperature, the rate of dye uptake on dyeable spandex was significantly higher than that of ordinary spandex, and the difference was more obvious in deep color. Thus, temperature has a greater impact on ordinary spandex during the dying process.

##### Thermodynamic Model and Calculation of Related Parameters

Based on the adsorption isotherms of spandex fibers in Figure 11 and Figure 12, the Langmuir and Freundlich models were used to study the experimental results in order to comprehensively analyze the adsorption properties of neutral red G on spandex.

**Figure 13:**
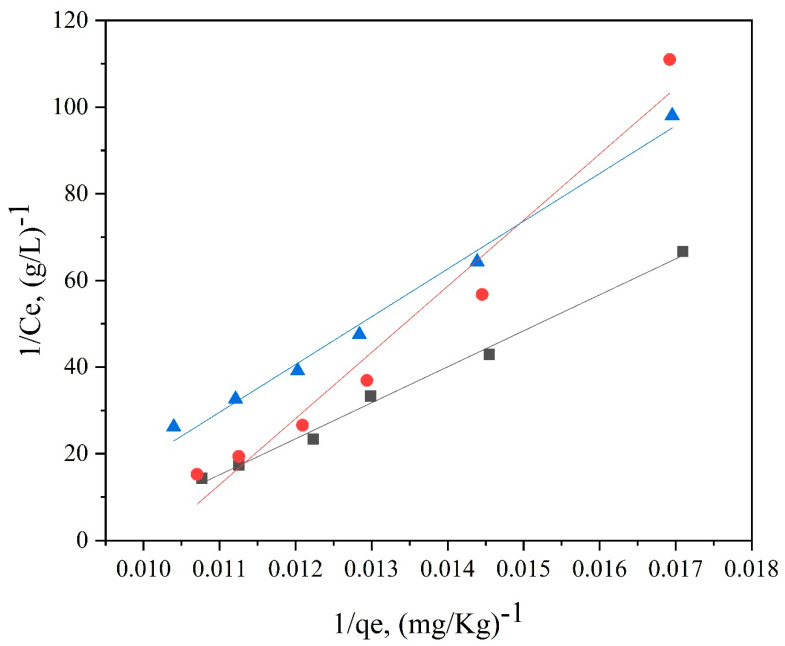
Langmuir model of dyeable spandex dyed with neutral red G.

**Figure 14:**
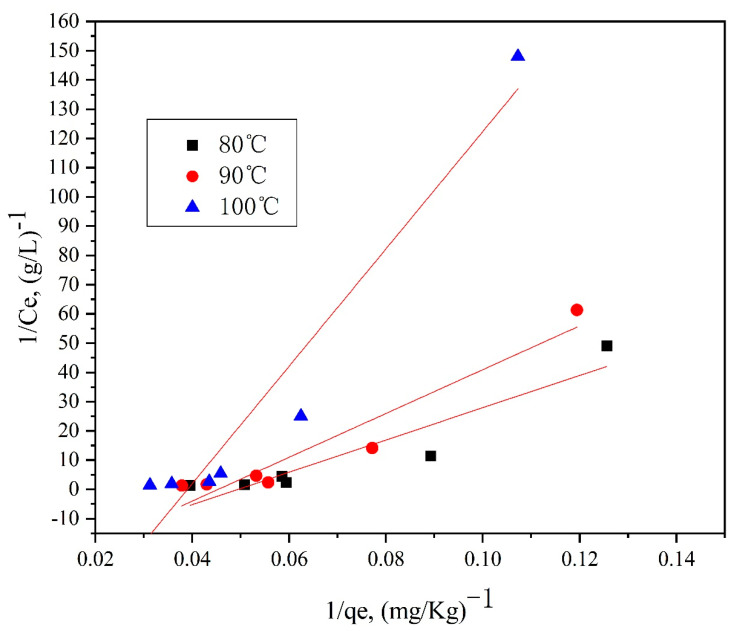
Langmuir model of ordinary spandex dyed with neutral red G.

**Figure 15:**
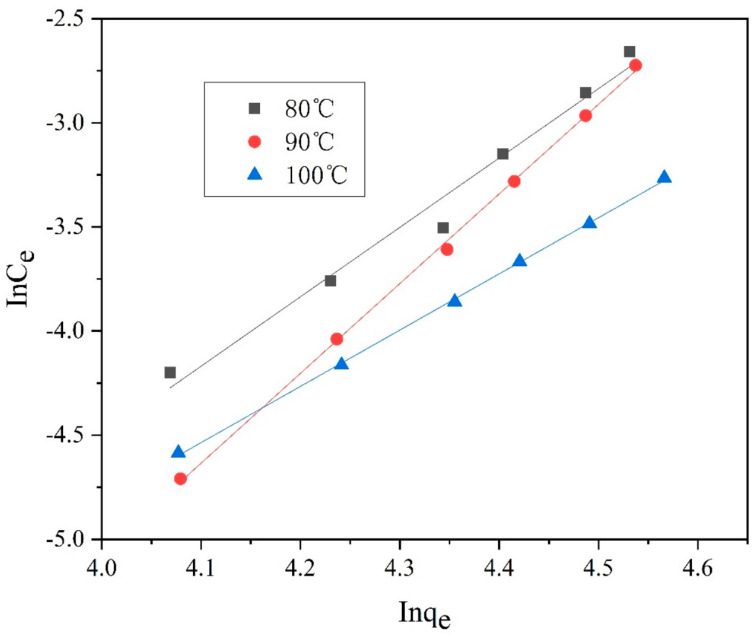
Freundlich model of dyeable spandex dyed with neutral red G.

**Figure 16:**
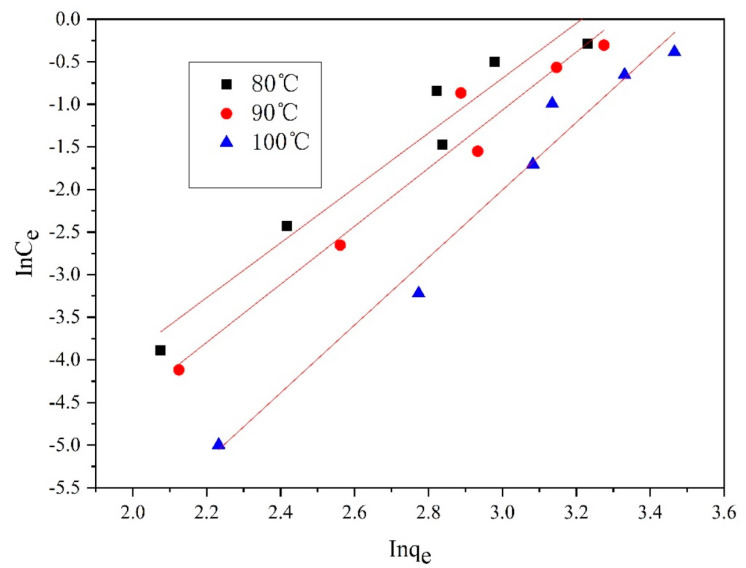
Freundlich model of the ordinary spandex dyed with neutral red G.

Furthermore, Figure 13, Figure 14, Figure 15 and Figure 16 depict the thermodynamic models of dyeable spandex and ordinary spandex fibers dyed on neutral red G at 80, 90, and 100 °C.

According to the adsorption isotherm model in Figure 13, Figure 14, Figure 15 and Figure 16, and calculation per Formulas (7)–(12), the thermodynamic parameters of dyeing can be calculated as shown in Table 3.

As shown in Table 4, the adsorption capacity (Q_f_) of dyeable spandex at 80, 90 and 100 °C for the Langmuir model were 110.65813 mg/g, 99.23536 mg/g and 121.26476 mg/g, respectively, which was similar to the adsorption capacity of gardenia yellow for wool under TCEP (118.9 mg/g). This result indicates that dyeable spandex has the same dye adsorption capacity as wool. However, the Q_f_ of ordinary spandex was only about 20 mg/g, which was low, so its adsorption capacity to dyes was poor. For the Freundlich model, the Q_f_ of dyeable spandex and ordinary spandex were both small, and the adsorption capacity of dyeable spandex was not consistent with the dyeing result. It indicates that the adsorption of neutral red G on dyeable spandex does not satisfy the Freundlich model.

As can be seen from Table 4, when the Langmuir model linearly fitted the dyeable spandex, the regression coefficient R^2^ was generally higher than that of the Freundlich model, indicating that the adsorption of neutral red G on dyeable spandex was more consistent with the Langmuir model. This is consistent with the results obtained in the kinetic study; dye adsorption on spandex is the chemical location adsorption. This is mainly because the dyeable spandex contains a large number of secondary amino groups, which can be bonded with dyes by ionic bonds under acidic conditions. For the ordinary spandex, the regression coefficient R^2^ was higher when Freundlich model was used, indicating that the adsorption mechanism of dyes on the ordinary spandex was more dependent on Vander Waals force and hydrogen bond, which was mainly due to the lack of dye blocks such as dyeable spandex.

**Figure 17:**
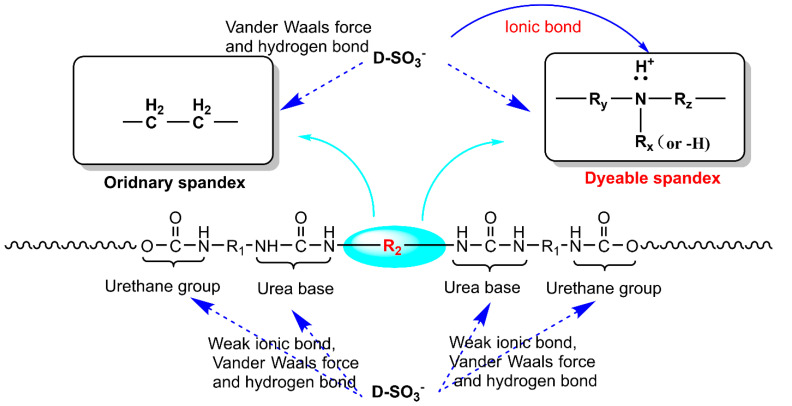
Adsorption mechanism of neutral red G on spandex.

Figure 17 depicts the adsorption mechanism of neutral red G on spandex based on dyeing thermodynamics and kinetics results. The adsorption of neutral dye on the ordinary spandex was mainly through Vander Waals force and hydrogen bond. In addition, nitrogen atoms of ordinary polyurethane fibers in urea and carbamate groups could be bonded with dyes by ionic bonds. Due to the action of highly electronegative oxygen atoms in urea and carbamate groups, the formed ionic bonds were very weak. Otherwise, this part of the molecular alignment was high, the adsorption capacity of the dye was less; The primary factor that makes spandex dyeable is that the nitrogen atoms in the chain extender were positively charged by combining with hydrogen protons under acidic conditions, and the negative ions of sulfonate with neutral dyes were bonded by ionic bonds. At the same time, there were a certain amount of Vander Waals forces and hydrogen bonds. However, the chain extender of the ordinary spandex consisted of common carbon atoms, which lacked the dye base for dye adsorption. It could only rely on Vander Waals force and hydrogen bonds to combine with the dye, resulting in a weak binding force and low adsorption capacity. In addition, the chain extender of the dyeable spandex used mixed amines, which were different from ethylenediamine of the ordinary spandex. The introduction of these mixed amines reduced the linear regularity of spandex macromolecules and increased the accessibility of dyes.

Table 5 illustrates the thermodynamic parameters of spandex dyed with neutral red G. When △G ° was negative, dye adsorption to fiber was spontaneous, and dyeing affinity was inverse. The affinities of dyeable spandex and ordinary spandex dyed with neutral red G were greater than zero and increased with temperature rise. In addition, the dyeing affinity of dyeable spandex was greater than that of ordinary spandex, which may be due to amino groups present in dyeable spandex. Under acidic conditions, dyestuffs bound to the amino group of the spandex through ionic bonds, and neutral dyes, had greater directness to dyeable spandex. Thus, neutral dyestuffs were more likely to be absorbed into dyeable spandex.

Dyeing heat, △H°, represents heat change in the dyeing process. It refers to the heat generated during the transfer of 1 mol of acid dye from the dye bath to spandex in a standard state [19,20,21,22]. If it is greater than zero, the dyeing process is endothermic; otherwise, it is exothermic. Table 5 reveals that it was less than zero, indicating that dyeing of dyeable spandex and ordinary spandex was an endothermic reaction. Moreover, it was easier for neutral red G to react with dyeable spandex because the energy barrier between the reaction of dyeable spandex and neutral dyestuffs was lower, and the binding trend between dyeable spandex and dyestuffs was relatively strong.

The dyeing entropy △S° indicates the change in the degree of disorder of the system caused by dyestuffs’ transfer from solution to fiber. For example, when dyeable spandex was dyed with neutral red G, △S° was positive, indicating that the degree of disorder in the system had increased. However, when the neutral red G was transferred to dyeable spandex, the dyestuffs became disorganized. Thus, it must be rearranged onto the fibers.

## 4. Conclusions

This study investigated the adsorption mechanism of neutral red G on dyeable spandex. The research concluded that, compared with ordinary spandex, dyeable spandex used mixed amines as chain extenders, which reduced the regularity of molecules and increased the accessibility of anions. At the same time, a large number of secondary amino groups provided reaction sites for anionic adsorption, which strengthened the adsorption characteristics of ion location and significantly increased the equilibrium adsorption capacity of dyeable spandex to anion. The adsorption of neutral red G on dyeable spandex satisfied the quasi-second-order kinetic model and Langmuir thermodynamic model. The research results can provide an important reference for the application and development of dyeable spandex in dyeing and anionic-supported materials.

## Figures and Tables

**Table 1:** Kinetic model parameters of spandex dyeing with neutral red G.

	Pseudo–First-Order Kinetic			Pseudo–Second-Order Kinetic			Time of Adsorption Dyeing t_1/2_/min
	k_1_/min^−1^	q_e_/mg × g^−1^	R^2^	k_2_K_2_ g/mg × min	q_e_/mg × g^−1^	R^2^	
Dyeable spandex	0.4052	19.5007	0.8481	0.1497	19.5007	0.9998	0.3424
Ordinary spandex	0.0266	10.9745	0.9645	0.0043	10.9745	0.9689	20.2853

**Table 2:** Comparison of equilibrium adsorption capacity of different fibers.

Samples	q_e_/mg × g^−1^	Dyestuff
Dyeable spandex	19.50078	Neutral red G
Ordinary spandex	10.9745	Neutral red G
Wool fiber [26]	24.08	Gardenia yellow
Nylon 6 [27]	15.6006	Madder
Cotton [28]	18.85	Gardenia yellow

**Table 3:** Diffusion coefficient of spandex dyed with neutral red G.

Type of Spandex	Dyeable Spandex	Ordinary Spandex
Diffusion coefficient D/10^−10^	9.06336	0.153

**Table 4:** Adsorption isotherm model parameters of spandex dyed with neutral red G.

Type of Spandex and Temperature		Langmuir Mode			Freundlich Mode		
		Q_f_	b	R^2^	Q_f_	*n*	R^2^
		(g/kg Spandex)	(g/L)^−1^		(g/kg Spandex)		
Dyeable spandex	80 °C	110.65813	75.01575	0.99217	1.00908	3.22252	0.93426
	90 °C	99.23536	159.1314	0.95485	1.01013	3.41012	0.94745
	100 °C	121.26476	91.65565	0.98768	1.00828	3.96926	0.96991
Ordinary spandex	80 °C	20.25685	27.2557	0.84311	1.0506	3.30582	0.94147
	90 °C	22.07199	33.92804	0.90125	1.04635	4.32015	0.9974
	100 °C	25.6401	78.26255	0.92051	1.03977	2.67926	0.99896

**Table 5:** Thermodynamic parameters of spandex dyed with neutral red G.

Type of Spandex and Temperature		B/(g/L)^−1^	−△G°/KJ	△H°/KJ	△S°/J × K^−1^
Dyeable spandex	80 °C	75.01575	12.67715	10.97467	0.004820831
	90 °C	159.1314	15.30668		0.011928975
	100 °C	91.65565	14.01662		0.008152093
Ordinary spandex	80 °C	27.2557	9.70455	57.78239	−0.136140009
	90 °C	33.92804	10.64049		−0.129813842
	100 °C	78.26255	13.52654		−0.118600686

## Data Availability

The date presented in this study are available on request from the corresponding author.
